# Males but not females report genital sensations evoked by fixed-parameter stimulation of somatosensory cortex

**DOI:** 10.1093/brain/awaf240

**Published:** 2025-07-03

**Authors:** Sandra Proelss, Mehmet S Tuncer, Christine Heim, John-Dylan Haynes, Peter Vajkoczy, Michael Brecht, Katharina Faust

**Affiliations:** Bernstein Center for Computational Neuroscience Berlin, Charité – Universitätsmedizin Berlin, corporate member of Freie Universität Berlin and Humboldt Universität zu Berlin, Berlin 10117, Germany; Institute of Biology, Humboldt-Universität zu Berlin, Berlin 10117, Germany; NeuroCure Cluster of Excellence, Charité–Universitätsmedizin Berlin, Berlin 10117, Germany; Department of Neurosurgery, Charité-Universitätsmedizin Berlin, Corporate Member of Freie Universität Berlin and Humboldt Universität zu Berlin, Berlin 10117, Germany; Institute of Medical Psychology, Charité–Universitätsmedizin Berlin, Corporate Member of Freie Universität Berlin and Humboldt-Universität zu Berlin, Berlin 10117, Germany; Bernstein Center for Computational Neuroscience Berlin, Charité – Universitätsmedizin Berlin, corporate member of Freie Universität Berlin and Humboldt Universität zu Berlin, Berlin 10117, Germany; Berlin Center for Advanced Neuroimaging, Charité – Universitätsmedizin Berlin, corporate member of Freie Universität Berlin and Humboldt Universität zu Berlin, Berlin 10117, Germany; Department of Neurosurgery, Charité-Universitätsmedizin Berlin, Corporate Member of Freie Universität Berlin and Humboldt Universität zu Berlin, Berlin 10117, Germany; Bernstein Center for Computational Neuroscience Berlin, Charité – Universitätsmedizin Berlin, corporate member of Freie Universität Berlin and Humboldt Universität zu Berlin, Berlin 10117, Germany; Institute of Biology, Humboldt-Universität zu Berlin, Berlin 10117, Germany; NeuroCure Cluster of Excellence, Charité–Universitätsmedizin Berlin, Berlin 10117, Germany; Department of Neurosurgery, Charité-Universitätsmedizin Berlin, Corporate Member of Freie Universität Berlin and Humboldt Universität zu Berlin, Berlin 10117, Germany

**Keywords:** somatotopy, sex differences, intraoperative mapping, body image, postcentral gyrus, somatosensory cortex

## Abstract

The localization of the human genital cortex has been debated since its unusual placement in Wilder Penfield’s somatosensory homunculus. While male and female genitalia are different, it remains unclear how these external differences are mapped onto the male and female brain.

We investigated genital representation in the human somatosensory cortex by patient-report of sensations evoked by fixed parameter electrical stimulation during awake craniotomies.

We find a reproducible genital representation in male subjects (*n* = 3) at the somatotopically appropriate location between the legs situated in the dorsal postcentral gyrus and sulcus. Our findings contradict early stimulation maps derived by Penfield and colleagues, which indicated an absence of genital responses in this brain region, but align with more recent imaging data from males and females that described blood-flow responses to genital stimulation at these coordinates. Surprisingly, however, we find no evidence for stimulation-evoked genital sensations in the postcentral gyrus and sulcus of females (*n* = 5) in line with Penfield’s earlier conclusions. Specifically, females reported no genital sensations, but often leg sensations, when stimulated at the putative coordinates of female genital cortex.

We conclude that reports of genital sensations differ between male and female somatosensory cortex upon stimulation. Our observations add to the growing evidence that genital representations differ between males and females.

## Teaser

Proelss *et al.* describe the mapping of human genital representations by electrical stimulation of somatosensory cortex. Males but not females report genital sensations in response to stimulation of the postcentral gyrus, adding to evidence that genital representations differ between males and females.

## Introduction

**Figure 1:**
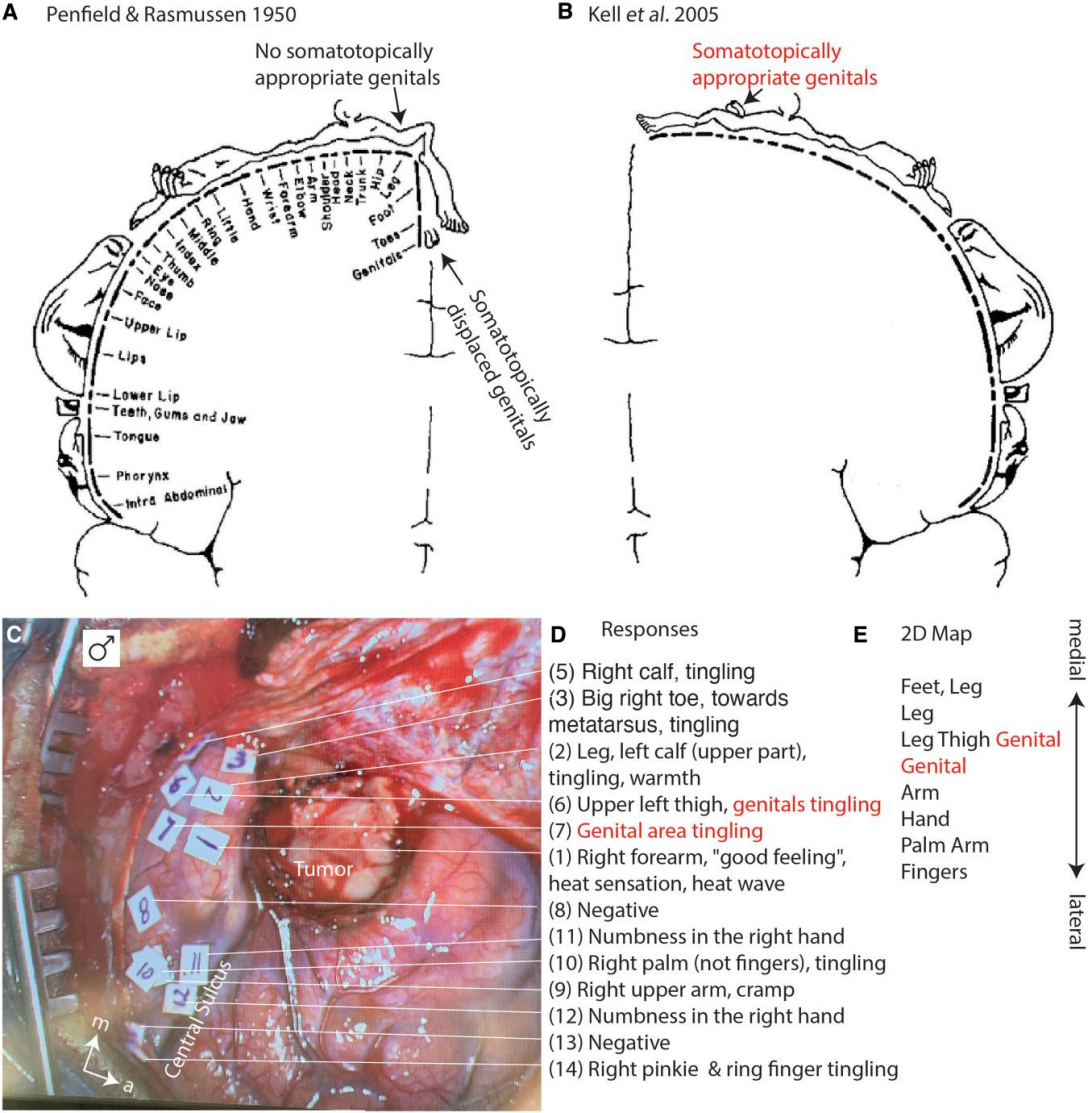
**Dorsal somatosensory cortex body maps without and with genitals at somatotopic appropriate locations and a cortical map of a male subject with genital sensations**. (**A**) The classic somatosensory cortical map from Penfield and Rasmussen^2^; note the absence of somatotopically appropriate genitals in dorsal somatosensory cortex. (**B**) Somatosensory cortical map from Kell *et al*.^4^ with a somatotopically appropriate genital representation in dorsal somatosensory cortex. (**C**) Intrasurgical photograph of the craniotomy over the right cortical hemisphere of a 28-year-old male. Numbers indicate stimulation points (50 Hz, bipolar, 0.8 ms, 8 mA). m = medial, l = lateral. (**D**) Stimulation evoked sensory responses. Note the report of genital sensations (stimulation points 6 and 7). (**E**) 2D map of responses. Note the presence of somatotopically appropriate genital sensations (genitals indicated between leg/thigh and arm).

Thinking about cortical body representations was shaped by the seminal work of Wilder Penfield and colleagues.^1,2^ These somatosensory maps artistically translated into homunculi by Hortense Cantlie^3^ captured neuroscientists. Most aspects of the Penfield maps are undisputed, but the genital representation suggested by Penfield and colleagues is still debated. Specifically, Penfield suggested a displaced location of the human genitals below the feet (Fig. 1A). This displaced-genitals suggestion contradicted the strict somatotopy and left the dorsal postcentral primary somatosensory cortex (S1)-body representation devoid of genitals. This absence of male genitals from the dorsal S1-body representation has since been challenged. Kell *et al*.^4^ showed responses to tactile penis stimulation in the S1-body representation with functional MRI (fMRI; Fig. 1B). Importantly, a recent fMRI study also showed female genital responses in dorsal postcentral gyrus.^5,6^ This reassessment of the human S1-genitalia representation aligns with physiological^7^ and neuroanatomical^8^ results that unequivocally show somatotopic S1 genital maps.

Other unusual characteristics of the S1 genital representation found less attention. Cortical genital maps of penis and clitoris in rodents are monomorphic, even though penis and clitoris differ markedly.^8,9^ The S1-genital-representation shows an unusual plasticity: in rodents, a detailed body map is apparent in cortical layer 4.^10^ This body map is apparent at birth, when it still can be altered, but is pretty much frozen (immutable) 1 week later, even if dramatic interventions are applied.^11^ The layer 4 map of genitals is the exception to this immutability rule. The layer 4 map of genitals expands in puberty,^8^ as a result of genital touch^12^ and sexual experience.^13^ The frequency of sexual intercourse also appears to positively affect the thickness of the female human genital cortex.^5^ Experience of childhood sexual abuse, in contrast, seems to have a destructive effect on human genital cortex and reduces cortical thickness in this region.^14^

We stimulated the exposed cortex of awake human patients and asked: (i) Do males report electrically evoked genital sensations and, if so, where? (ii) Do females report genital sensations, and, if so, where? (iii) What do stimulation-evoked genital sensations tell us about male-female differences?

## Materials and methods

See the Supplementary material for additional methodological detail.

### Ethics

The study was approved by the local ethics committee (Charité–Universitätsmedizin Berlin; EA1/019/18), in accordance with the Declaration of Helsinki. Written informed consent was obtained from all study participants.

### Patient selection

Eight patients (five female, three male) with infiltratively growing tumours within the central region and in proximity to the mantle fold were enrolled prospectively.

### Surgical workflow

All surgeries were performed under local anaesthesia. A customary scalp block was applied, numbing the respective branches of the trigeminal and cervical nerves using a mixture of bupivacaine 0.5% and epinephrine (ratio 250.000:1).

### Direct cortical stimulation protocol

For direct cortical stimulation, a bipolar stimulation probe with a pole distance of 10 mm was operated through an intraoperative neurostimulation system (ISIS^®^, Inomed^®^ Medizintechnik GmbH). A stimulation intensity of 6–8 mA, impulse width of 200 µs, frequency of 50 Hz, stimulus number of 1 and positive pulse form were used as standard stimulation parameters. The stimulation threshold was determined as the minimum current necessary to evoke a sensory response. This current strength was employed throughout the remainder of the testing protocol.

### Data recording and normalization

During intraoperative mapping, the stimulation probe was linked to a navigation probe.

### Intraoperative patient interview

During awake testing, the experiment involved stimulation of the cortical surface area. Following each stimulation, the experimenter engaged the patient in a dialogue to ascertain whether any sensations or feelings were experienced.

### MRI

All patients underwent MRI in a 3-T (Siemens Sykra) scanner.

### Statistical analyses

We used Fisher’s exact test to compare the distribution of genital versus non-genital sensations between sexes. We also compared the incidence of genital responses with an unpaired *t*-test. We modelled the effects of tumour size and responsiveness using a generalized linear model (GLM).

## Results

### Male body map

We mapped human somatosensory cortex during tumour surgeries. Patients underwent craniotomy under local anaesthesia. Sedation was discontinued once the procedure was completed, and awake patients described the sensations produced by electrical stimulation (50 Hz, bipolar, 0.8 ms, 6–8 mA currents). We tailored patient inclusion and stimulation procedures for mapping the dorsal postcentral gyrus.

We first report a mapping experiment in a male subject. As for the other patients, excellent stimulation coverage of the dorsal postcentral gyrus was achieved (Fig. 1C). The patient reported numerous electrically evoked bodily sensations (Fig. 1D), including genital sensations. Reported sensations formed an orderly map of the body surface (Fig. 1E), in which genital sensations mapped somatotopically appropriately next to the upper leg and thigh. Two other mapping experiments in males yielded similar results. Males reported genital sensations at somatotopically appropriate positions in the dorsolateral postcentral gyrus. In the two additional male subjects, the genital cortex extended into the central sulcus and could have been overlooked if we had not specifically searched for genital responses. Reports of genital sensations were reproducible when the points were stimulated again. Our mapping results in males contradict the Penfield maps (Fig. 1A) but align with the imaging results (Fig. 1B).^4^

### Female body map

**Figure 2:**
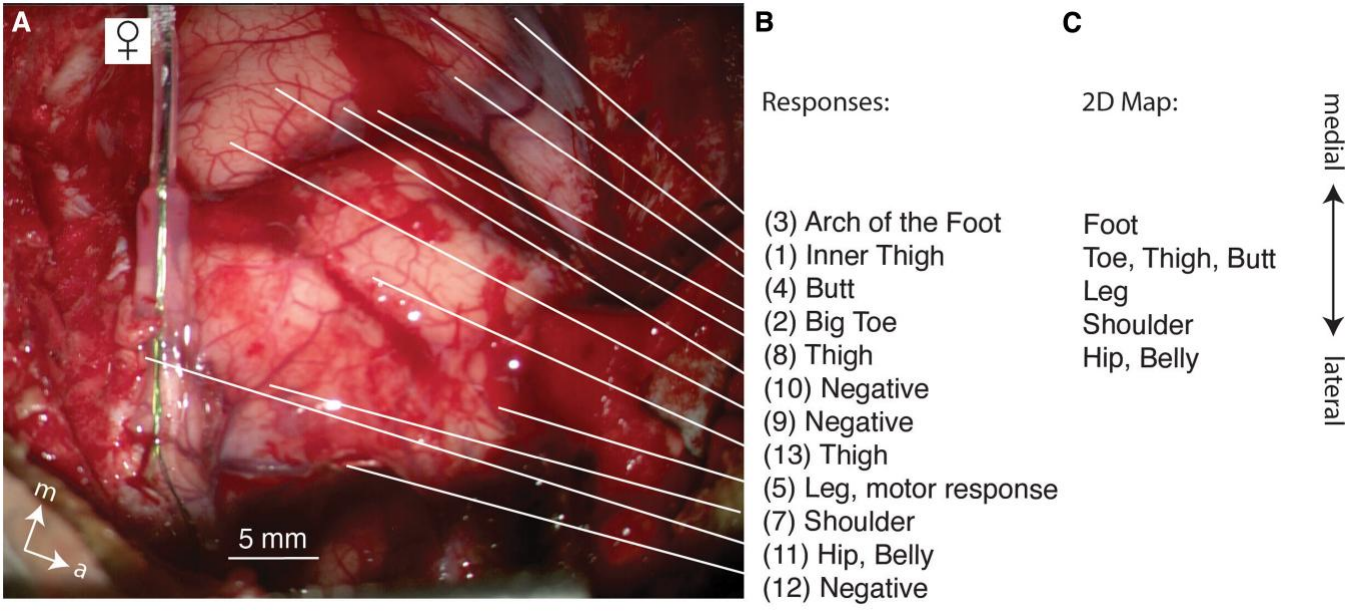
**A cortical map from female postcentral gyrus without genital sensations.** (**A**) Intrasurgical photograph of the craniotomy over the left cortical hemisphere of a 50-year-old female. The image is a still from a video taken through the surgery microscope. The image was taken after the mapping procedure. We show a *post hoc* picture, because the numbers placed on the cortical surface occluded the exposure. We mirror-imaged the photograph to simplify the alignment of the results with the right-hemispheric experiment shown in Fig. 1. The left ends of the white lines indicate the stimulation points (50 Hz, bipolar, 0.8 ms, 6–8 mA). (**B**) Stimulation evoked sensory responses. (**C**) 2D map of responses. Note the absence of genital sensations.

Next, we report a mapping experiment in a female subject (Fig. 2). Again, we obtained excellent coverage of the postcentral gyrus (Fig. 2A). The female patient reported numerous somatic sensations in response to electric stimulation (Fig. 2B). The sensations did not include genital sensations, however. We observed an orderly somatotopic map of somatic sensations not including the genitals (Fig. 2C). Similar observations were made in four additional patients. Females reported somatic but not genital sensations, despite thorough exploration of the dorsal postcentral gyrus.

### Comparison of male and female responses

**Figure 3:**
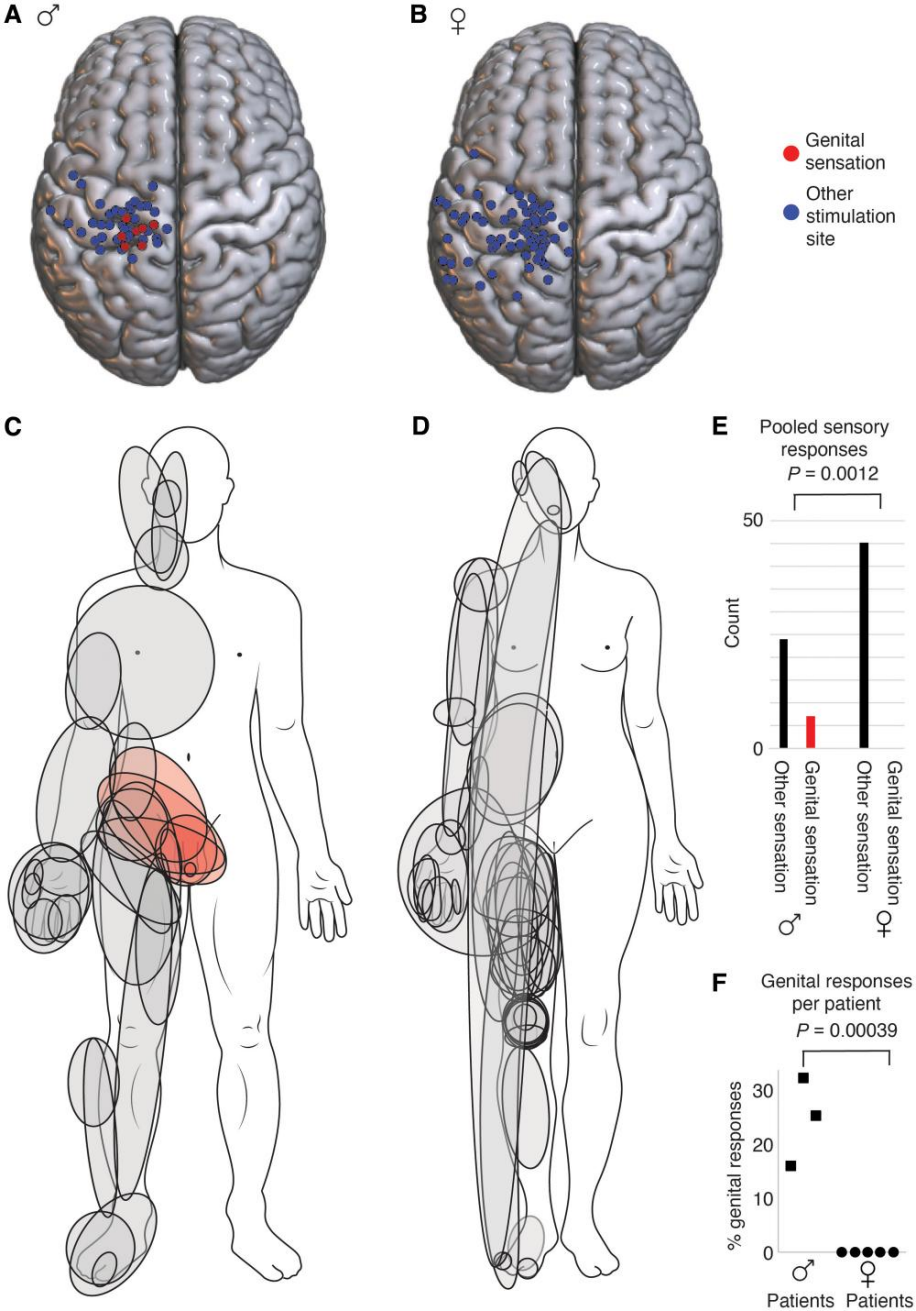
**Males but not females report electrically evoked genital sensations in response to stimulation of the postcentral sulcus.** (**A**) Composite (*n* = 3 males) map of stimulation points superimposed to a standard brain (MNI space). Note the prominent cluster of points where stimulation evoked genital sensations (red). (**B**) Composite (*n* = 5 females) map of stimulation points and responses; conventions as in **A**; note the excellent coverage of the brain surface and absence of evoked genital sensations. (**C**) Depiction of stimulation evoked tactile sensations verbally reported by males on a drawing of a male body. Note the complete coverage of the body. (**D**) Depiction of stimulation evoked tactile sensations verbally reported by females on a drawing of a female body. Note the omission of the genitals. (**E**) Population plot of stimulation-evoked sensations according to stimulation site responses reported in males (7 genital and 24 non-genital sensations) and females (0 genital and 45 non-genital sensations). The distributions are significantly different; the *P*-value refers to a Fisher’s exact test. (**F**) Population plot of stimulation-evoked sensations according patients. The incidence of genital responses (plotted as per cent of genital sensations of all evoked sensations) reported in three males and five females. The incidence of genital responses was significantly different between males and females; the *P*-value refers to an unpaired *t*-test. MNI = Montreal Neurological Institute.

Individual cases suggested male-female differences. To pool across subjects, we transferred the stimulation points into Montreal Neurological Institute (MNI) space.^15^ We show the pooled stimulation points and associated patient responses for males in Fig. 3A. The pooled data showed a relatively even stimulation coverage of cortex around the dorsal central sulcus. Genital sensations mapped to very similar positions, enforcing the idea that the reports reflected the activation of male genital cortex across subjects. A similar pooled map of stimulation points and responses for females is shown in Fig. 3B. Stimulation coverage was excellent, but no genital sensations were reported. To visualize how the male body is represented in the dorsal postcentral gyrus, we depict verbally reported sensations graphically in Fig. 3C. As expected from the even stimulation point coverage, the male body is completely represented in the reported responses. The male genitals were well represented in parts with small response fields. In females, somatic responses also covered the body, but the genitals were omitted (Fig. 3D). As shown in a population analysis using Fisher’s exact test in Fig. 3E, the distribution of genital and non-genital sensations differed significantly between males and females (*P* = 0.0012). We also plotted the incidence of genital responses patient-by-patient (Fig. 3F), which was significantly higher in male than female patients (*P* = 0.00039; unpaired *t*-test).

### Responses evoked at putative genital cortex coordinates

**Figure 4:**
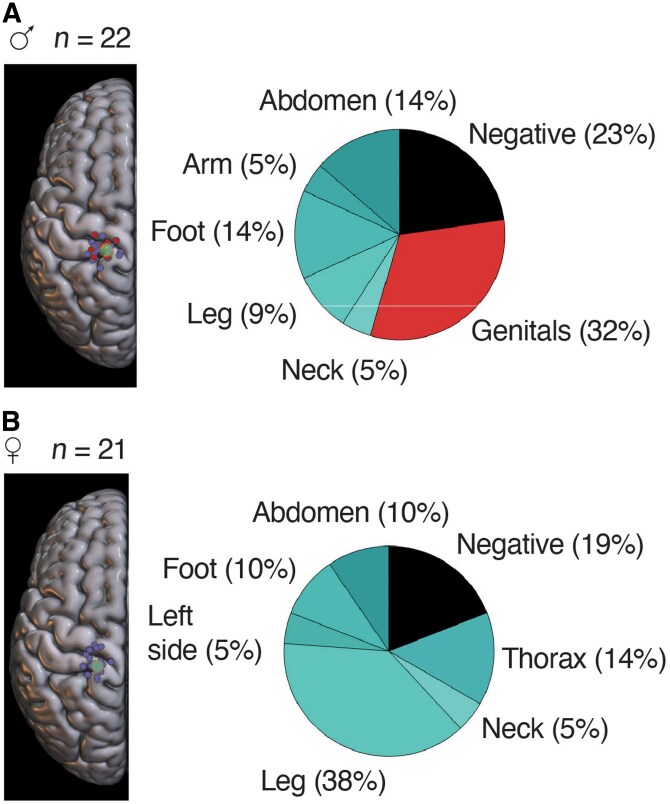
**Stimulation responses evoked at the coordinates of female genital cortex as identified by MRI of sensory genital responses**. (**A**) *Left*: Composite map of stimulation points in males superimposed to a standard brain (MNI space). Only stimulation points close to the coordinates of female genital cortex (the centre of which is marked by the green disk) as identified by imaging of sensory responses in females by Knop *et al*.^5^ are shown. As indicated by *n*, we placed numerous stimulation points at such coordinates. Stimulation points where stimulation evoked genital sensations are shown in red; other stimulation points are shown in blue. *Right*: Pie chart of sensory responses evoked in males at cortical coordinates of female genital cortex. Note the large fraction of genital sensations, which indicates that male and female genital cortex share the same cortical coordinates. (**B**) *Left*: Composite map of stimulation points in females superimposed to a standard brain (MNI Space). Only stimulation points close to the coordinates of female genital cortex (the centre of which is marked by the green disk) as identified by imaging of sensory responses in females by Knop *et al*.^5^ are shown. As indicated by *n*, we placed numerous stimulation points at such coordinates. Conventions as in **A**. *Right*: Pie chart of sensory responses evoked in females at cortical coordinates of female genital cortex. Note the absence of genital sensations and the large fraction of leg sensations. The fraction of negative responses is slightly lower than in males, suggesting female genital cortex is not simply unresponsive; instead, genital cortex stimulation seems often to evoke leg sensations. MNI = Montreal Neurological Institute.

We wondered which sensations—if any—were evoked by stimulation of the genital cortex in males and females. To this end, we separately plotted stimulation sites close to or at the centre of female genital cortex as identified by us in an earlier imaging study.^5^ Figure 4A, left shows a collection of stimulation sites at these female genital cortex coordinates. Numerous sites were stimulated at these coordinates in males. Males often reported genital sensations at such sites (Fig. 4A, right), suggesting that male genital cortex is located at the same coordinates as female genital cortex. In females, we also stimulated numerous sites at the coordinates of female genital cortex (Fig. 4B, left). Females did not report genital sensations at such sites (Fig. 4B, right). Interestingly, the fraction of negative responses at such coordinates was even slightly lower than in males, suggesting that female genital cortex is not simply unresponsive. Instead, females conspicuously often reported leg sensations at genital cortex stimulation sites (Fig. 4B, right). We concluded that females report different sensations from males in response to fixed-parameter stimulation of their genital cortex.

### Cortical responsiveness

Since genital responses were only observed in male patients—a surprising finding—we tested the influence of tumour size, sex and age on stimulation response likelihood using a GLM with logistic regression (Supplementary Table 1). None of these covariates showed a statistically significant association with response likelihood. Specifically, the model estimated a negative but non-significant association for tumour size [*β* = −0.305, standard error (SE) = 0.235, *P* = 0.194, 95% confidence interval (CI) −0.765, 0.155] and age (*β* = −0.019, SE = 0.027, *P* = 0.475, 95% CI −0.072, 0.033), while male sex had a small positive estimated effect (*β* = 0.305, SE = 0.850, *P* = 0.720, 95% CI −1.361, 1.971). The overall model fit was not statistically significant compared to a constant-only model (*χ*^2^ = 5.41, *P* = 0.144).

When assessing the effect of tumour type on response likelihood, the logistic regression model revealed a statistically significant overall effect (*χ*^2^ = 9.68, *P* = 0.046) of histology. This was primarily driven by a single (female) patient with radiation-associated necrosis and significantly reduced response probability (*β* = −1.282, SE = 0.506, *P* = 0.011, 95% CI −2.274, −0.290) compared to astrocytoma. All other tumour types did not differ significantly from astrocytoma: glioblastoma (*β* = 0.253, SE = 0.568, *P* = 0.656, 95% CI −0.860, 1.366), meningioma (*β* = −0.594, SE = 0.581, *P* = 0.307, 95% CI −1.733, 0.545) and oligodendroglioma (*β* = −0.114, SE = 0.672, *P* = 0.865, 95% CI −1.431, 1.203).

Taken together, these findings suggested that tumour histology and patient and disease characteristics do not affect overall stimulation responsiveness. However, the small sample size limited the statistical power of these analyses and increased the risk of a type II error. Future studies and larger sample sizes are needed to assess these covariates properly.

## Discussion

### Presence and location of male genital cortex

Several lines of argument indicate that we have identified male genital cortex: (i) genital sensations were evoked in all males; (ii) stimulation thresholds were low; (iii) genital sensations co-localized across subjects (who were unaware of the location of the stimulation sites); (iv) genital sensations were evoked at coordinates where fMRI work had identified responses to penis stimulation^4^; and (v) the responses mapped to a somatotopically appropriate position. Accordingly, Penfield and colleagues’ idea of an absence of genital sensations in dorsal postcentral cortex is no longer tenable. Two factors may account for differences between our work and the mapping studies by Penfield *et al*. First, we were aware from our own and other people’s work that genital sensory responses are evoked in the dorsal postcentral gyrus. Second, unlike Penfield and colleagues, we searched for genital cortex.

### The absence of stimulation evoked genital reports in female postcentral gyrus

For several reasons, we regard the absence of genital responses in the postcentral gyrus of females with our stimulation parameters—albeit unexpected—to be a correct result: (i) in all five female subjects, no genital sensation were observed; (ii) in contrast, females readily reported other somatic sensations; (iii) the absence of genital sensations aligns with Penfield and colleagues^1^ and may have triggered Penfield’s unusual genital cortex suggestion; and (iv) in rats, motor responses can be evoked from somatosensory cortex at similar thresholds in males and females, with the exception of genital somatosensory cortex, where stimulation thresholds for genital movements are several times higher in females.^16^ Thus, sex differences specifically in genital cortex are not unprecedented. Human female genital cortex localization via fMRI previously placed it within the postcentral gyrus, at a site thoroughly stimulated in our study. Male patients reported genital sensations at this site upon stimulation, while females largely described leg sensations. Thus, the female and male genital cortex share a cortical location but differ in stimulation-evoked reports. Because we used fixed stimulation parameters, we cannot rule out the possibility that the genital cortex has higher current thresholds in females. A ‘higher genital cortex current thresholds in females’ hypothesis might explain the absence of genital sensations elicited by cortical stimulation, even though imaging experiments can identify female genital cortex.^5^

Early evidence has already hinted at sex differences in the S1 genital cortex threshold: Penfield and Boldrey^1^ noted ‘evidence is not sufficient for conclusion’ on female genital placement, because stimulation failed to elicit genital reports. In contrast, men’s genital representation could be identified, albeit with difficulty.

Sex differences in peripheral receptors and their neural pathways might indirectly affect cortical thresholds by influencing how genital sensations are integrated centrally. For example, recent research shows that mouse clitoris has a 15-fold higher density of Krause’s end-bulb corpuscles (specialized for detecting vibration and light-touch) compared to the penis.^17^ Furthermore, genital tactile signals are exclusively processed in a specialized spinal cord region distinct from those processing other touch signals. This functional segregation of sensory input could enable specific sensory gating mechanisms that modulate genital signals, potentially affecting cortical thresholds for genitals and not for other body areas. While both sexes receive dense genital innervation via the pudendal nerve, the presence of additional, distinct sensory pathways in females (such as vagal afferents innervating internal structures that bypass spinal relay circuits)^18^ might further influence cortical sensory integration.

### Modulatory influences on somatosensory cortical responses and threshold

Sex differences in genital cortex stimulation threshold may not be fixed and may be subject to modulatory influences. Use-dependent plasticity suggests that infrequent sensory input could elevate cortical thresholds and potentially lead to cortical thinning, whereas regular sensory stimulation might lower thresholds by expanding cortical representation. For example, cortical thickness within the female genital S1 region correlates positively with the frequency of sexual activity.^5^ Conversely, genital numbing—due to sexual trauma, dysfunction or medication effects—may lead to decreased cortical responsiveness.^14,19^

Hormonal modulation, particularly by ovarian steroids such as oestrogen, is another potential contributor to variability in cortical responsiveness. Oestrogen levels fluctuate across menstrual cycles and reproductive stages^6^ and are linked to changes in somatosensory cortex representation. For example, lactating rodents show an expanded S1 representation of the ventral trunk and nipple areas.^20^ Similarly, the age-related decline in oestrogen during menopause might directly affect cortical representation and responsiveness of genital regions.^21,22^ Such hormonal influences across a woman’s lifespan (menstrual cycles, pregnancy, lactation, menopause) could modulate somatosensory signals. While our study did not collect specific data on menopause or hormonal status, previous research indicates that age alone generally does not affect cortical stimulation thresholds for other body areas.^23^ Thus, the modulatory influences discussed here likely reflect hormonal and experience-dependent changes rather than simply age-related effects.

Lesion characteristics and tumour-induced plasticity are known to influence stimulation effects and thresholds. Slow-growing pathologies in particular (e.g. low-grade glioma) have been reported to induce cortical reorganization. For example, a tumour in the primary motor cortex may drive the motor representation posteriorly across the central sulcus into S1,^24^ and similarly diffuse gliomas involving S1 have been observed to remap somatosensory functions to surrounding cortex.^25^ Tumour presence can modulate the thresholds and responses during stimulation mapping.^26^ While we found that tumour type or size had largely no effect on overall responsiveness in our study—making a change in excitability a less likely contributor in this context—our findings are based on a small sample size.

Genital cortex—an area not even acknowledged in many human^2^ and animal studies^8^—appears to be exceptionally interesting. Why do females report genital sensations less readily than males upon stimulation? When it comes to ultimate causes, evolutionary theory might provide answers. The evolution of asymmetrical parental investment, as posited by Triver’s^27^ parental investment theory, predicts that the sex investing less in offspring (typically males) show a higher degree of sexual responsiveness than females, who are associated with higher biological investment through gestation, nursing and menopause.^28^ Accordingly, we wonder if the differences in reported genital sensations reflect a larger sexual responsiveness in human males compared to females.

## Conclusion

We conclude that male genital cortex follows somatotopic organization and that genital reports evoked by cortical stimulation differ between males and females. Genital cortex stimulation appears to elicit genital responses in males and leg responses in females. Further work is needed to understand the functional implications of the absence of female genital reports.

## Supplementary Material

awaf240_Supplementary_Data

## Data Availability

We provide all data and figures under a G-node registry upon publication. A compressed version of the original images will be uploaded upon publication.
