# Supplementary material for: Males but not females report genital sensations evoked by fixed-parameter stimulation of somatosensory cortex
Source: Brain. 2025 Jul 3;148(11):3872–9. doi: 10.1093/brain/awaf240 (PMC12588675; doi:10.1093/brain/awaf240)
Supplement: awaf240_Supplementary_Data [file awaf240_supplementary_data.pdf]

Supplementary material

Males but not females report genital sensations evoked by fixed-parameter stimulation of somatosensory cortex

By Sandra Proelss, Mehmed S Tuncer, Christine Heim, John-Dylan Haynes, Peter Vajkoczy, Michael Brecht, Katharina Faust

Supplementary material is available at *Brain* online.

Patient selection

Eight patients (five female, three male) with infiltratively growing tumours within the central region and in proximity to the mantle fold were prospectively enrolled. Inclusion criteria were scheduled awake surgery for motor and sensory mapping and tumour location in the required area near the mantle fold. Exclusion criteria were: age < 18y, inability to give informed consent, cognitive compromise, preexisting motor or sensory deficit, seizure frequency of > 1 per week. Patients of both sexes were included. Tumours in both hemispheres were included.

Data from a sixth female patient were excluded, because the mapping in this case did not include the dorsal part of the S1 representation. The mapped area included parts of ventral S1, putative area S2 and insular cortex. This female reported numerous bodily sensations, but no genital sensations; the case aligns with the idea of a paucity of genital sensations evoked in the female brain (Supplementary Table 1).

Surgical workflow

All surgeries were performed under local anaesthesia. A customary scalp block was applied, numbing the respective branches of the trigeminal and cervical nerves using a mixture of bupivacaine 0.5% and epinephrine (ratio 250.000:1). Heads were fixed into a head clamp that allowed the surgeon sufficient tumour access while allowing the patient a comfortable position to communicate with the examiner. The skull surface was registered into a navigation system (Brainlab® cranial buzz navigation) later used for DCS stimulation registration. Navigated craniotomies were performed that allowed optimal exposure of the tumour, the pre- and postcentral gyri as well as the mantle edge. If necessary, small doses of systemic analgesia and sedation (propofol and remifentanil 2.5 mg/kg/hr) were administered during skull opening and wound closure. At least

20 minutes before awake testing, any sedation or systemic analgesia was stopped. All corticotomy was performed after completion of cortical mapping.

DCS protocol

Stimulation threshold was determined as minimum current necessary to evoke a sensory response. This current strength was employed throughout the remainder of the testing protocol. In order to both confirm effective functioning of the DCS and to anticipate epileptic seizures, electrocorticography (ECoG) was recorded via a 6-contact electrode strip placed at the margin of the craniotomy. Stimulation testing was performed when the patient was responsive, alert and in adequate absence of any sedative or systemic analgetic medication. In the area of the central and postcentral sulci the arachnoid layer was removed and the cortex of the sulcal valleys was mapped in analogous manner to the gyral mounds.

Data recording and normalisation

Each stimulation positive point was registered onto the patient's cortical surface and auto-registered into the patient's individual MPRAGE data set via the software "acquire point" function (Brainlab® Elements; Brainlab® AG, Munich, Germany). To each recorded stimulation point the specific neurological response was allocated (e.g.: "*stimulation point 4 - tingling in left upper medial thigh*"). In addition, numbered paper markers were placed for direct visualisation of the positive spots (Fig. 1C). Coordinates of stimulation points were converted into a dicom matrix, and were registered to the Montreal Neurological Institute 152 (MNI) space using FSL (https://fsl.fmrib.ox.ac.uk/fsl). The anatomical images were skull stripped prior to registration using optiBET.sh.[1,2]

Intraoperative patient interview and sham stimulation

The patients were encouraged to provide detailed and spontaneous descriptions of any perceived sensations. In cases where the patient had difficulty articulating the exact location of the sensation, a body outline allowed the patient to visually indicate where the sensation occurred. To verify the reliability of the sensations reported, stimulations that produced significant effects were repeated non-consecutively. Additionally, at points of interest, the experimenter conducted a sham stimulation, where no actual stimulation was applied, but the patient was led to believe that one had occurred. Sham stimulation usually evoked no sensations. In one exception, a patient reported tingling sensation in his left foot following a sham stimulation, and a fleeting tingling sensation around the navel, moving to the thigh, groin and scrotum before dissipating and re-emerging for around three minutes following another sham stimulation in absence of active testing.

Magnetic Resonance Imaging

All patients underwent MRI in a 3-T (Siemens Sykra) scanner. Contrast-enhanced, 1-mm slice thickness magnetization-prepared rapid gradient echo (MPRAGE) images (TR = 2300, TE = 2.32, TI = 900 ms, 9° flip angle, 256 × 256 matrix, 1 mm isotropic voxels, 192 slices) for neuronavigation mapping. Additional sequences were made for tumour visualisation and surgical planning.

Statistical Analyses

We conducted a Fisher's exact test based on the number of genital and non-genital responses reported by male and female patients to compare the distribution of genital versus non-genital sensations between sexes at the population level. We also compared the incidence of genital responses in males and females with an unpaired t-test.

To test whether the occurrence of genital responses was related to overall cortical responsiveness across patients, we modelled the proportion of trials that elicited any type of response (genital, other sensory responses, motor, or mixed motor/sensory responses combined) as a function of patient and disease characteristics. We selected a sequential modelling approach to maximise statistical power while acknowledging sample size constraints. First, we assessed the influence of continuous and binary covariates (tumour size, sex, and age) using a generalised linear model (GLM) with a binomial distribution and logit link function.

Next, the multi-level categorical predictor (tumour type) was analysed separately in a second binomial GLM to avoid overfitting the model, as inclusion of multiple categorical levels would have further reduced the available degrees of freedom. Model significance was assessed using the chi-square statistic comparing the fitted model to a constant model. All models were implemented in MATLAB using fitglm. Structures of both models can be found below.

Patient data

An overview of patient and tumour characteristics is presented in supplementary table 1. Stimulation responses show counts of genital, other sensory, motor and mixed (sensory and motor responses evoked by one stimulation), as well as negative responses (no stimulation effect was observed or reported). Patient 9 was excluded as the tumour was situated temporally and dorsal S1 was not accessible.

Model approach

We assessed whether patient and tumour characteristics were associated with overall cortical responsiveness using binomial generalized linear models (GLMs) with a logit link, implemented in MATLAB's fitglm function.

Multivariable GLM:

logit(Response Probability) = Intercept + Sex + Age + Tumour Size

Due to its multi-level categorical structure and small group sizes, tumour type was analysed separately in a univariate GLM:

Univariate GLM:

logit(Response Probability) = Intercept + Tumour Type

References

1. Jenkinson M, Beckmann CF, Behrens TE, Woolrich MW, Smith SM. FSL. *Neuroimage*. 2012;62:782-790.
2. Smith SM. Fast robust automated brain extraction. *Hum Brain Mapp*. 2002;17:143-155.

Supplementary Table 1: Patient characteristics

ID	Sex	Age	Tumour Type	Tumour Location	Tumour Size	Geni-tal	Other Sensory	Motor	Mixed	Nega-tive	Total N Stimulation Events	Res-ponses (%)
1	**M**	28	Astrocytoma	Postcentral (in S2)	Ca 4 cm	2	10	1	0	3	16	81
2	**M**	43	Astrocytoma	Central (M1/ S1)	Ca 5.2 cm	2	4	4	1	2	13	85
3	**M**	32	Astrocytoma	Central (M1/S1/ S2)	Ca 3.8 cm	3	7	3	2	4	19	79
4	**W**	45	Meningeoma	Extraaxial Postcentral (S1)	Ca 2.5 cm	0	10	2	1	7	20	65
5	**W**	67	Glioblastoma	Precentral Gyrus (M1)	Ca 2cm	0	6	6	0	4	16	75
6	**W**	50	Oligodendro-glioma	Precentral Gyrus (M1)	Ca 3.5 cm	0	12	2	0	2	16	88
7	**W**	58	Glioblastoma	Postcentral Gyrus (S1)	Ca. 3.6 cm	0	10	2	0	4	16	75
8	**W**	65	Radiation-associated Necrosis	Superior Parietal Lobule	Ca. 4.6 cm	0	5	8	1	15	29	48
(9)	W	42	Oligodendro-glioma	Postcentral (in S2)	Ca. 4.4 cm	0	14	5	1	10	30	67
